# Comparison of Highly and Weakly Virulent *Dickeya solani* Strains, With a View on the Pangenome and Panregulon of This Species

**DOI:** 10.3389/fmicb.2018.01940

**Published:** 2018-08-31

**Authors:** Malgorzata Golanowska, Marta Potrykus, Agata Motyka-Pomagruk, Michal Kabza, Giovanni Bacci, Marco Galardini, Marco Bazzicalupo, Izabela Makalowska, Kornelia Smalla, Alessio Mengoni, Nicole Hugouvieux-Cotte-Pattat, Ewa Lojkowska

**Affiliations:** ^1^Department of Biotechnology, Intercollegiate Faculty of Biotechnology, University of Gdansk and Medical University of Gdansk, Gdansk, Poland; ^2^Department of Integrative Genomics, Faculty of Biology, Adam Mickiewicz University, Poznan, Poland; ^3^Department of Biology, University of Florence, Florence, Italy; ^4^EMBL, EBI, Wellcome Trust Genome Campus, Cambridge, United Kingdom; ^5^Department of Epidemiology and Pathogen Diagnostics, Julius Kühn-Institut - Federal Research Centre for Cultivated Plants, Braunschweig, Germany; ^6^UMR5240 Microbiologie Adaptation et Pathogénie, Univ Lyon, CNRS, Univ Claude Bernard Lyon 1, INSA Lyon, Villeurbanne, France

**Keywords:** adhesin, genome comparison, *Pectobacteriaceae*, prophages, regulon comparison

## Abstract

Bacteria belonging to the genera *Dickeya* and *Pectobacterium* are responsible for significant economic losses in a wide variety of crops and ornamentals. During last years, increasing losses in potato production have been attributed to the appearance of *Dickeya solani*. The *D. solani* strains investigated so far share genetic homogeneity, although different virulence levels were observed among strains of various origins. The purpose of this study was to investigate the genetic traits possibly related to the diverse virulence levels by means of comparative genomics. First, we developed a new genome assembly pipeline which allowed us to complete the *D. solani* genomes. Four *de novo* sequenced and ten publicly available genomes were used to identify the structure of the *D. solani* pangenome, in which 74.8 and 25.2% of genes were grouped into the core and dispensable genome, respectively. For *D. solani* panregulon analysis, we performed a binding site prediction for four transcription factors, namely CRP, KdgR, PecS and Fur, to detect the regulons of these virulence regulators. Most of the *D. solani* potential virulence factors were predicted to belong to the accessory regulons of CRP, KdgR, and PecS. Thus, some differences in gene expression could exist between *D. solani* strains. The comparison between a highly and a low virulent strain, IFB0099 and IFB0223, respectively, disclosed only small differences between their genomes but significant differences in the production of virulence factors like pectinases, cellulases and proteases, and in their mobility. The *D. solani* strains also diverge in the number and size of prophages present in their genomes. Another relevant difference is the disruption of the adhesin gene *fhaB2* in the highly virulent strain. Strain IFB0223, which has a complete adhesin gene, is less mobile and less aggressive than IFB0099. This suggests that in this case, mobility rather than adherence is needed in order to trigger disease symptoms. This study highlights the utility of comparative genomics in predicting *D. solani* traits involved in the aggressiveness of this emerging plant pathogen.

## Introduction

Bacteria belonging to the genera *Dickeya* and *Pectobacterium* (formerly genus *Erwinia*, soft-rot *Enterobacteriaceae*), recently reclassified into the new family *Pectobacteriaceae* (Adeolu et al., 2016), have been responsible for significant economic losses in European crop production (Perombelon, 2002; Toth et al., 2011; Mansfield et al., 2012). These bacterial phytopathogens cause blackleg symptoms on potato and soft rot disease on many other host plants. Despite the fact that *D. solani* strains were described as one of the most aggressive blackleg and soft rot-causing bacteria, their mechanism of virulence has not been fully elucidated yet. The competitive advantage of *D. solani* relies on a wider range of temperatures favorable to disease development than in the case of other *Dickeya* spp., as well as on the fact that lower inoculum levels are sufficient for an effective infection spread (Czajkowski et al., 2013). Outside symptomatic plants, *D. solani* can also be found as saprophytic bacterium in rhizosphere soil (Heuer et al., 2010; Potrykus et al., 2014). However, different virulence levels were observed among *D. solani* isolates of various origins (Potrykus et al., 2014; Golanowska et al., 2017).

All of the *D. solani* strains investigated so far share genetic homogeneity. In more detail, *dnaX, dnaN, fusA, gapA, gyrA, purA, rplB, rpoS, recA*, and 16S rDNA sequences of *D. solani* showed 100% identity in Multilocus Sequence Analysis (MLSA) (Slawiak et al., 2009; van der Wolf et al., 2014; Potrykus et al., 2016). Likewise, no genomic differences were shown by classical DNA fingerprinting methods, i.e., REP PCR and PFGE, even though the analyzed strains originated from various European countries (Slawiak et al., 2009; Degefu et al., 2013; van der Wolf et al., 2014; Potrykus et al., 2016; Golanowska et al., 2017). Similar outcomes resulted from the variable number of tandem repeats (VNTR) method differentiating only 3 patterns for 54 *D. solani* isolates (Parkinson et al., 2014). Such a low genetic variation was attributed to a limited number of introductions and/or a recent emergence of *D. solani* in Europe.

The species *D. solani* is closely related to the well described species *Dickeya dadantii*. The virulence of *D. dadantii* relies on the coordinated production of high levels of multiple secreted enzymes, including pectinases, cellulases and proteases, which breakdown the plant cell wall and release nutrients used for bacterial growth (Barras et al., 1994; Hugouvieux-Cotte-Pattat et al., 1996; Py et al., 1998; Thomson et al., 1999; Perombelon, 2002; Hugouvieux-Cotte-Pattat, 2016). Analyses of *D. solani* genomes revealed the existence of additional genes potentially involved in pathogenicity and production of toxins, including clusters that encode polyketide synthases (PKS), non-ribosomal peptide synthetases (NRPS), amino acid adenylation domain, and proteins transported via T5SS/T6SS (Garlant et al., 2013; Pédron et al., 2014).

Efficiency to attach to the plant organs and cell motility can also influence virulence (Khayi et al., 2015; Weller-Stuart et al., 2017). It is usually considered that the ability of bacteria to attach to plant tissue increases its aggressiveness (Nair et al., 2003; Liao et al., 2014; Chen et al., 2017). However, some studies demonstrated inverse effects. For instance, a highly virulent mutant of *Xylella fastidiosa* has lower attachment to the xylem vessels than the wild type strain (Gottig et al., 2009; Ionescu et al., 2014). The authors concluded that strong attachment of bacteria to plant surfaces could restrict their movement, thus capacity to colonize the plants and finally limit disease severity.

The involvement of prophages in disease symptoms was demonstrated in different plant pathogens, such as *Pectobacterium, Pseudomonas, Ralstonia* and *Streptomyces* (Varani et al., 2013). Phages and prophages can affect the bacterial genome in many different ways for instance by gene disruption or shuffling and affect expression of adjacent genes. By introducing new fitness factors, including pathogenicity determinants, phages can even cause an avirulent strain to become virulent (Varani et al., 2013).

The appropriate regulation of genes encoding virulence factors is essential for setting off pathogenesis. It was first shown in *D. dadantii* 3937 that a set of transcriptional factors (TF), acting as global or specific regulators, enables bacterial adaptation during the infection process (Hugouvieux-Cotte-Pattat, 2016; Reverchon et al., 2016). The combined action of the regulators KdgR, CRP, Fur, PecS, and PecT, plays a decisive role in the proper synchronization of virulence factors production. The KdgR regulator triggers induction of pectinases after the pathogen have sensed the presence of pectin, a major component of middle lamellae, which is a part of plant cell wall. CRP (catabolite activator protein) is a highly conserved global regulator, whose function is to direct bacteria toward the utilization of preferential carbon sources, depending on the nutrient availability (Hugouvieux-Cotte-Pattat, 2016). The iron-dependent repressor Fur (ferric uptake regulator) is necessary to control iron uptake and iron homeostasis (Franza et al., 1999, 2002). The global regulators PecS and PecT play an essential role in the switch from epiphytic to pathogenic lifestyle, by preventing the premature expression of genes encoding virulence factors (Reverchon et al., 2016). Potrykus et al. (2014) showed that the regulators KdgR, PecS, and PecT have similar functions in *D. solani* as in *D. dadantii*.

Apart from genomic homogeneity stated for *D. solani* strains, our former studies (Potrykus et al., 2014, 2016, 2018) showed significant differences between various isolates in their ability to cause disease symptoms on diverse plant hosts, such as potato or chicory. Most of all, the highest virulence level was attributed to the strains originating from diseased plants in comparison to the strains isolated from the rhizosphere of healthy potatoes. The goal of the present study was to extensively describe the variability of the genomes of *D. solani* strains isolated from various origins, in search for genetic signatures possibly related to diverse virulence of these strains. To our knowledge, this is the first work providing an insight into pangenome and panregulon of the economically important plant pathogen *D. solani*.

## Materials and methods

### *Dickeya solani* strains and genomic sequences

*D. solani* strains exhibiting different levels of virulence were included in this work (Table 1). Two strains were isolated in Poland from rotten potato plants and showed a high level of virulence on potato tubers and chicory leaves (IFB0099, IFB0158), while 2 other isolates originated from healthy potato rhizosphere in Germany (IFB0221, IFB0223), showing either intermediate or low virulence on potato tubers and chicory leaves. The genomes of these 4 strains were assembled and annotated in our laboratory. The draft genomic sequence of IFB0099 had been published previously, with the GenBank accession no. JXRS00000000 (Golanowska et al., 2015). The version discussed here has an accession no. CP024711. Three other genome sequences were deposited in GenBank with the following accession numbers: PENA00000000, PEMZ00000000 and CP024710, for IFB0158, IFB0221 and IFB0223, respectively. In addition, 10 *D. solani* strains isolated in different countries, whose genomes were sequenced and deposited in GenBank, were enclosed for pangenome and panregulon analyses, including the *D. solani* Type strain - IPO 2222 (NCPPB4479^T^, LMG25993^T^) and the strains GBBC 2040, MK16, MK10, D s0432-1, RNS 08.23.3.1A, RNS 05.1.2A, RNS 07.7.3B, PPO 9019, and PPO 9134 (origin and references in Table 1).

**Table 1:** Bacterial strains used in this study and their genomic features.

***D. solani* strain**	**Country, year of isolation, source**	**No. of scaffolds**	**No. of N bases**	**Genome size (bp)**	**% GC**	**GenBank accession (Reference)**
IFB0099	Poland, 2005, potato stem	1	0	4,932,920	56.24	CP024711 (this work)
IFB0158	Poland, 2009, potato	37	395	4,879,070	56.24	PENA00000000 (this work)
IFB0221	Germany, 2005, potato rhizosphere	38	394	4,878,255	56.24	PEMZ00000000 (this work)
IFB0223	Germany, 2005, potato rhizosphere	1	0	4,937,554	56.24	CP024710 (this work)
NCPPB4479^T^ IPO2222	The Netherlands, 2007, potato	1	9,200	4,867,258	56.22	AONU01000000 (Pritchard et al., 2013)
GBBC 2040	Belgium, 2007, potato	1	27,548	4,860,047	56.34	AONX01000000 (Pritchard et al., 2013)
MK10	Israel, potato	3	3,800	4,935,237	56.21	AOOP01000000 (Pritchard et al., 2013)
MK16	Scotland, river water	3	2,100	4,870,382	56.23	AOOQ01000000 (Pritchard et al., 2013)
D s0432-1	Finland, 2004, potato stem	4	0	4,904,518	56.20	AMWE01000000 (Garlant et al., 2013)
RNS 08.23.3.1A Dsl 3337	France, 2008, potato	1	12,124	4,923,743	56.25	AMYI01000000 (Khayi et al., 2014)
PPO 9019	The Netherlands, 2006, grape hyacinth	24	30	4,866,823	56.25	JWLS01000000 (Khayi et al., 2015)
PPO 9134	The Netherlands, 2008, hyacinth	22	187	4,870,830	56.24	JWLT01000000 (Khayi et al., 2015)
RNS 05.1.2A	France, 2005, potato	37	0	4,985,571	56.13	JWMJ01000000 (Khayi et al., 2015)
RNS 07.7.3B	France, 2007, potato	24	325	4,871,815	56.24	JWLR01000000 (Khayi et al., 2015)

For the phenotypic analysis, we used 9 strains of *D. solani* that were available at Intercollegiate Faculty of Biotechnology University of Gdansk and Medical University of Gdansk at the time of performing experiments: IFB0099, IFB0158, IFB0221, IFB0223, IPO 2222, GBBC 2040, MK16, D s0432-1 and RNS 08.23.3.1A. All the strains were grown at 30°C on crystal violet pectate medium (CVP) (Helias et al., 2012), Luria broth agar (LA), or in Luria broth (LB) (Lennox, 1955) for 24–48 h, unless otherwise stated. Liquid cultures were agitated at 200 rpm.

### Potato tuber maceration assay

Potato tubers (cv. Caesar) were inoculated with the tested *D. solani* strains as described previously (Hugouvieux-Cotte-Pattat, 2004). Briefly, potato tubers were washed in tap water and dried. Ten potato tubers, each containing a small hole made with a sterile pipette tip, were inoculated with 10 μl of the bacterial suspension (~5 × 10^8^ CFU ml^−1^). Inoculated holes were then covered with mineral oil to provide anaerobic conditions. After 48 h of incubation at 30°C under high (99%) humidity, the rotten potato tissue was removed and weighted. The experiment was performed three times.

### Quantitative determination of pectate lyase activity

The pectate lyase activity was measured in liquid bacterial cultures grown for 24 h at 30°C with agitation 200 rpm in M63 minimal medium (Miller, 1992), supplemented with 2 g l^−1^ glycerol for noninduced conditions, and with both 2 g l^−1^ glycerol and 2 g l^−1^ polygalacturonic acid (PGA, Sigma) for induced conditions. The pectate lyase activity was determined spectrophotometrically by monitoring (Specord, Analytic Jena) the formation of unsaturated products from polygalacturonate at 235 nm (Tardy et al., 1997). The pectate lyase specific activity is expressed as micromoles of unsaturated products liberated per minute per milligram of bacterial dry weight (μmol min^−1^ mg^−1^). The experiment was performed three times with three replicates.

### Plate assays for cellulase and protease activities

The ability to produce cellulases was analyzed on M63 agar plates supplemented with 2 g l^−1^ glycerol and 10 g l^−1^ carboxymethylcellulose (CMC). Two microliter of bacterial suspension containing 10^8^ CFU ml^−1^ were spotted onto this medium and incubated for 24 h at 30°C. Afterwards, the plates were flooded with 10 mg ml^−1^ of Congo Red solution for 10 min and subsequently washed for 5 min with 1 M NaCl (Wood, 1980). Protease production was detected on LA medium containing skim milk (12.5 g l^−1^) after incubation for 24 h at 30°C (Ji et al., 1987). For both tests, diameters of the clear halo zones appearing around the colonies were measured. All experiments were performed three times with three replicates.

### Plate assay for swimming ability

The bacterial ability to swim was tested on LA medium solidified by a low amount of agar (3 g l^−1^). The plates were stabbed with 0.2 μl inoculation loop. After incubation for 24 h at 30°C, the diameters of the bacteria spreading zones were measured. The experiment was performed once with three replicates.

### Statistical analysis

In order to analyze the significance of the phenotypic results, we utilized statistical computing and graphics R 3.3.1 (GNU project) programming environment. To check whether the data follow normal distribution, the Shapiro–Wilk test was used. Levene's test was implemented to verify whether the data variances are equal. As the requirements of ANOVA were not fulfilled, Kruskal–Wallis test was applied for multiple comparisons (R agricolae package) followed by *post-hoc* test using the Fisher's least significant difference criterion. All statistical hypotheses were tested at *p* < 0.05.

### Whole-genome shotgun sequencing and establishment of the genome assembly pipeline

**Figure 1:**
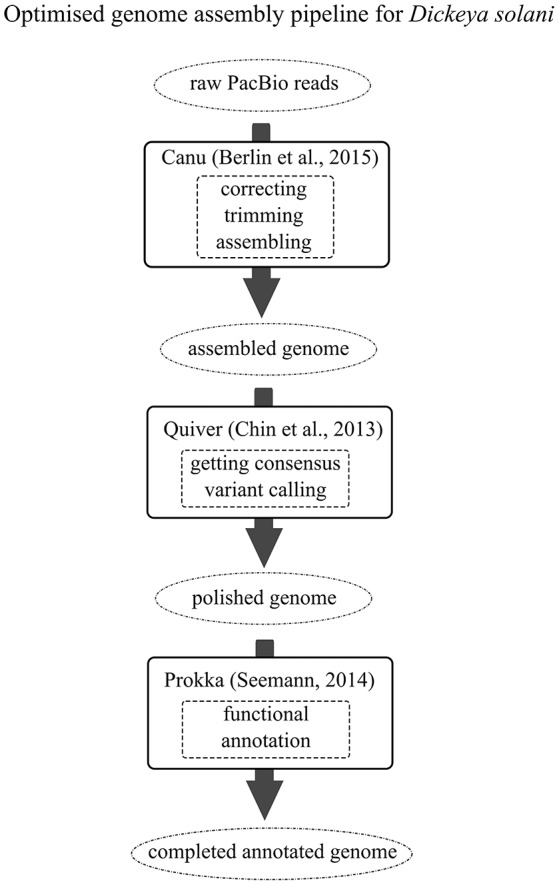
The optimized genome assembly pipeline for *D. solani*. Black arrows indicate the genomic assembly pipeline to follow. Different completeness stages of the constructed genome are presented within the dotted circles. Black boxes include data on the open source software and the corresponding literature references. Functions provided by certain software are enclosed within the dashed boxes.

Reads for the genomic sequences of strains IFB0099, IFB0158, IFB0221 and IFB0223 were acquired from BaseClear (The Netherlands). Different sequencing technologies and assembly methods have been tested in order to propose an optimized genome assembly pipeline leading to closing the genomes of *D. solani*. Regarding the first approach applied to the strains IFB0158 and IFB0221, respectively, 5,654,985 and 3,330,639 reads generated by MiSeq paired-end Illumina have been cleared out from adapters and low quality bases with the use of Trimmomatic (Bolger et al., 2014) (parameters: -phred33 LEADING:20 TRAILING:20 SLIDINGWINDOW:5:20 MINLEN:100). The application of SPAdes run together with MismatchCorrector (Bankevich et al., 2012) resulted in assembling IFB0158 and IFB0221 genomes into accordingly 37 or 38 scaffolds with ~395 N bases (Table 1). The IFB0099 and IFB0223 genomes were sequenced with PacBio platform (BaseClear, The Netherlands). 118,344 and 102,248 PacBio reads of IFB0099 (the previously reported draft genome of IFB0099 consisted of 97 scaffolds; Golanowska et al., 2015) and IFB0223 have been corrected, trimmed and assembled by using Canu (Berlin et al., 2015) (parameters: -pacbio-raw minOverlapLength = 500). The final genome polishing including getting consensus and variant calling was achieved by utilizing Quiver with the default settings (Chin et al., 2013). The above-proposed genome assembly pipeline (Figure 1) resulted in closing the genomes of IFB0099 and IFB0223 (with no N bases) by utilizing the reads derived solely from PacBio SMRT technology. All 14 genomes of *D. solani* strains used in this study were annotated by Prokka v1.11 (Seemann, 2014) for assuring uniformity of subsequent predictions. All the annotations of non-coding or regulatory RNAs yielded by Prokka's dependencies have been manually edited to meet the Genbank submission requirements. The genome of *D. dadantii* 3937 (Glasner et al., 2011) was utilized throughout as a reference.

### Bioinformatics analyses

#### General genome analyses

Average Nucleotide Identity (ANI) values were calculated for the available *D. solani* genomes by applying ChunLab's online Average Nucleotide Identity Calculator (EzBioCloud) (Yoon et al., 2017). This tool is based on OrthoANI algorithm, improved by using USEARCH instead of BLAST (Lee et al., 2016). The *D. dadantii* 3937 genome was used as an interspecies reference.

Synteny analysis was performed on genomes successfully closed to only one scaffold (IFB0099, IFB0223, IPO 2222, GBBC 2040, and RNS 08.23.3.1A) with the use of progressive Mauve available in Mauve 2.4.0 (development snapshot Mauve_2015_02_26) and the default settings (Darling et al., 2010). In this analysis, the IFB0099 genome was used as a reference.

The orthology assessment was performed by Mauve (Darling et al., 2004). Annotated genomes of 14 strains were aligned by Mauve and the orthological relationships between genes were extracted using the default parameters (min. 60% sequence identity, min. 70% alignment coverage). Each annotated gene was classified within the pangenome structure comprising the core (present in all analyzed genomes), accessory (present in more than a single genome, but not all) or unique (present in only one genome) pangenome fraction.

For protein motif prediction, the protein structures and the presence of signal peptides were analyzed at http://www.ebi.ac.uk/Tools/hmmer/, by utilizing hmmscan against Pfam database (HmmerWeb version 2.15.0; Finn et al., 2015).

The presence of prophages within the *D. solani* genomes was predicted by PHAST on-line web server (http://phast.wishartlab.com/). The completeness of the prophages and their classification as intact, questionable, or incomplete, which is linked with their potential viability, was assigned basing on the score calculations as described previously (Zhou et al., 2011).

#### Regulon and panregulon analyses

Regulon predictions were conducted on the basis of experimentally established and previously described sequences of the binding sites for four TFs: KdgR, Fur, CRP, and PecS (de Lorenzo et al., 1987; Sauvage et al., 1996; Rodionov et al., 2004; Rouanet et al., 2004; Franza et al., 2005). Regulon predictions have been performed with the use of MAST (version 4.10.1.,) (Bailey and Elkan, 1994) and *Bio.motifs* package from Biopython library (version 1.62b) (Cock et al., 2009) as reported previously (Galardini et al., 2015). In-house built Python (version 1.65, Cock et al., 2009) scripts were used for this purpose and are available here: https://github.com/combogenomics/regtools. Basically, the MAST suite was used for computing the k-mer frequencies for each genome (*fasta-get-markov* command) with a maximum k-mer length of 3 bp.

Regulatory motifs were searched in all the genomes by using the two algorithms reported above and retaining only concordant prediction obtained with both of them. Each regulatory motif correctly predicted has been parsed by grouping all the hits present in the upstream region of the putative target gene (up to 400 bp from the first codon). The panregulon was defined as the sum of all putative targets for a given TF in all the genomes considered (Galardini et al., 2011). The panregulon was subsequently divided into three parts—the core regulon (the set of targets for a given TF shared by all the tested genomes), accessory regulon (the set of targets found in not all but more than one genome) and unique regulon (targets found in only one genome). Similarity between strains that was based on panregulon analyses was represented by the UPGMA methods on Jaccard distance.

## Results

### Phenotypic comparison of the *D. solani* strains

**Figure 2:**
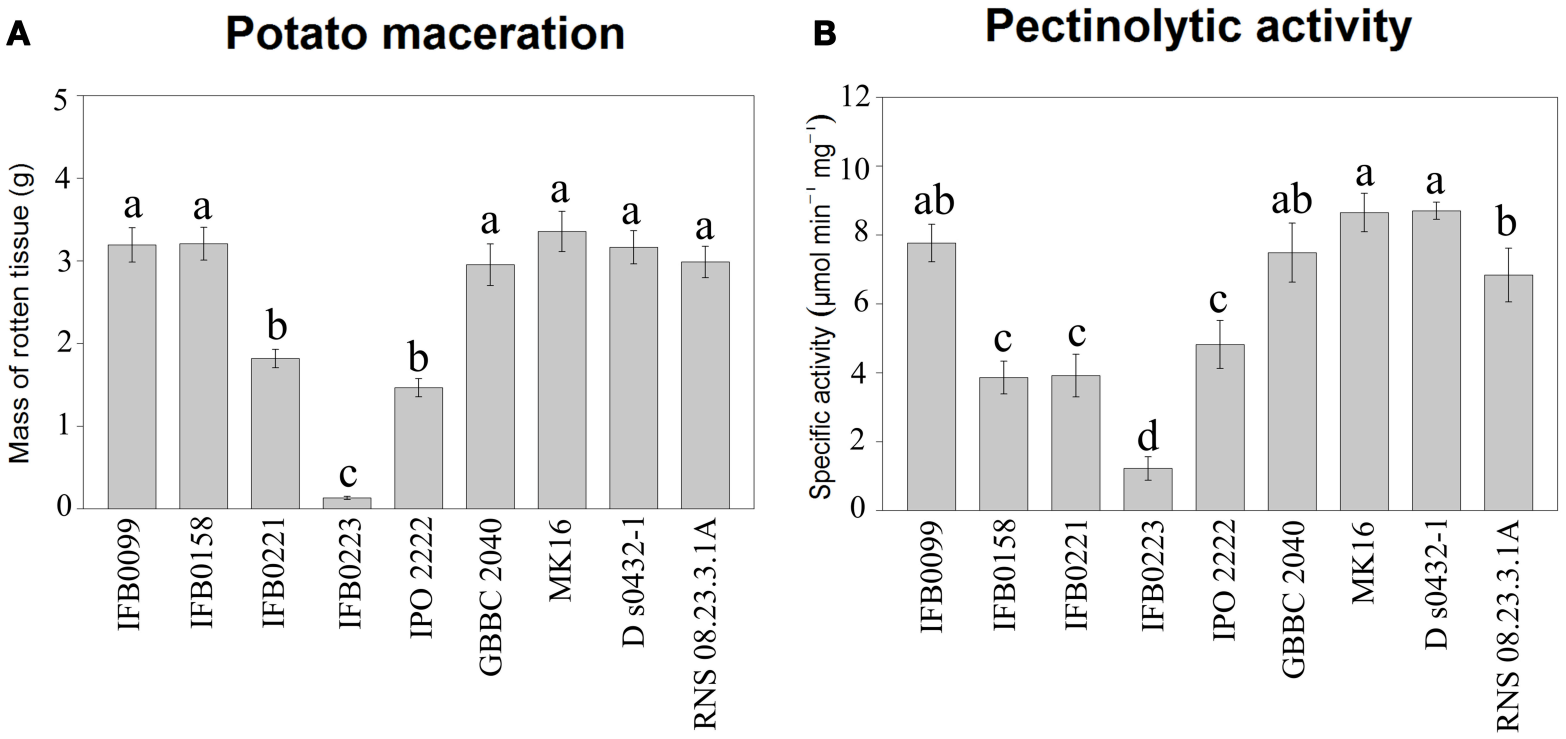
Potato tuber tissue maceration ability and pectinolytic activity of the *D. solani* strains. **(A)** Maceration ability in grams of macerated tissue, 48 h post inoculation. **(B)** Total pectate lyase specific activity (μmol min−1 mg−1) in the presence of polygalacturonic acid. Means ± standard errors are depicted, *n* = 10 for **(A)** and *n* = 3 for **(B)**. Within each panel mean values marked with different letters are significantly different by Kruskal–Wallis test followed by *post hoc* analysis using Fisher's least significant difference criterion (R agricolae package). All statistical hypotheses were tested at *p* < 0.05.

Several phenotypic features were determined for 9 *D. solani* strains available in our collection and whose genomes have been sequenced (IFB0099, IFB0158, IFB0221, IFB0223, IPO 2222, GBBC 2040, MK16, RNS 08.23.3.1A, and D s0432-1). The virulence of these *D. solani* strains, measured as the ability to macerate potato tissue, was compared. We distinguished three groups of strains on the basis of their virulence: high, intermediate and low virulent strains. High virulence was exhibited by IFB0099, IFB0158, GBBC 2040, MK16, RNS 08.23.3.1A, and D s0432-1. Strains IFB0221 and IPO 2222 showed intermediate virulence with maceration decreased by 53 and 46%, respectively, in comparison to IFB0099. The lowest virulence level was observed for the strain IFB0223, with a 96% reduction in comparison to IFB0099 (Figure 2A). The differences between the high, intermediate and low virulent strains were statistically significant (Figure 2A).

**Figure 3:**
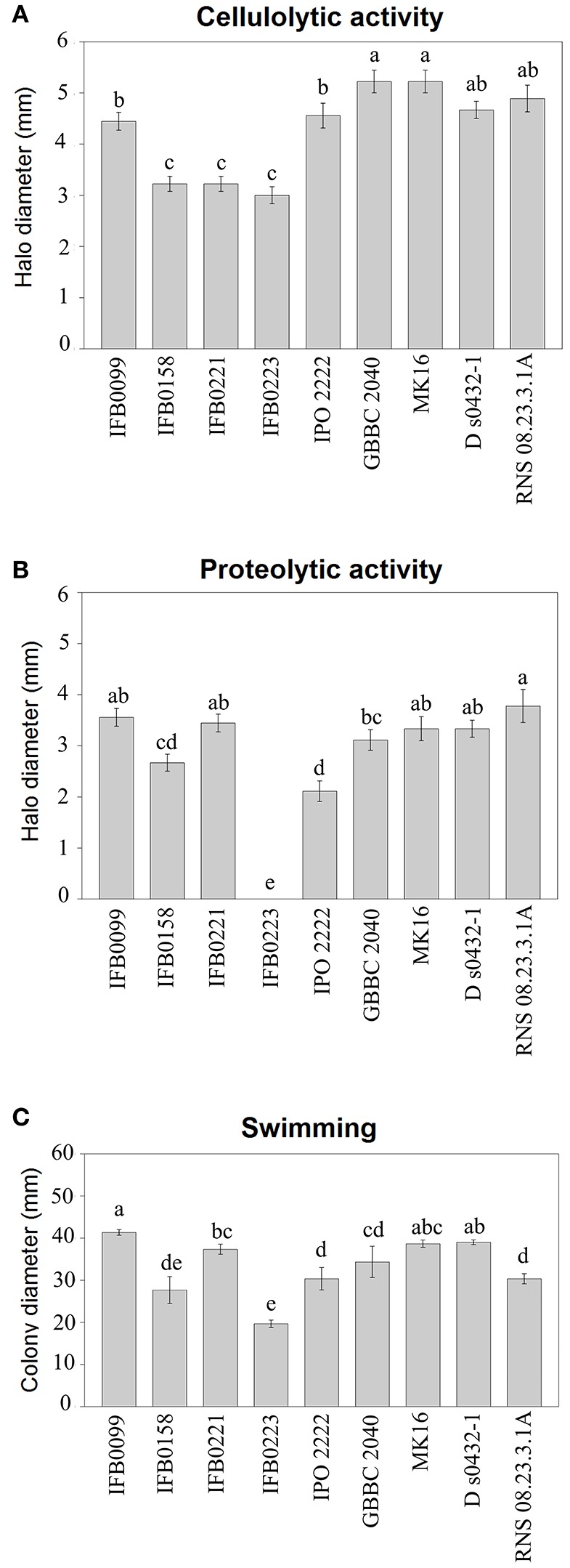
Phenotypic characterization of *D. solani* strains. **(A)** Cellulase production was estimated by the diameter (mm) of the haloes observed on the detection plates. **(B)** Protease production was estimated by the diameter (mm) of the haloes observed on the detection plates. **(C)** Motility was estimated by the diameter (mm) of the spread of the colonies in the low amount of agar (3 g l^−1^) medium. All the experiments were performed three times. Means ± standard errors are depicted, *n* = 3. Within each panel, mean values marked with different letters are significantly different by Kruskal–Wallis test followed by *post hoc* analysis using Fisher's least significant difference criterion (R agricolae package). All statistical hypotheses were tested at *p* < 0.05.

The activity of pectate lyase, being one of the main virulence factors, was analyzed. In noninduced conditions, all the *D. solani* strains showed a low pectate lyase activity (lower than 0.5 μmol min^−1^ mg^−1^) (data not shown). In induced conditions, IFB0099, GBBC 2040, MK16, D s0432-1, and RNS 08.23.3.1A strains showed the highest pectate lyase activity, from 7.7 to 8.7 μmol min^−1^ mg^−1^. Strains IFB0158, IFB0221, and IPO 2222 exhibited an intermediate pectate lyase activity, from 3.9 to 4.8 μmol min^−1^ mg^−1^. The lowest pectate lyase activity of ~1.3 μmol min^−1^ mg^−1^ was observed for the strain IFB0223 (Figure 2B). For the cellulase activity, two groups were differentiated (Figure 3A). Six strains (IFB0099, IPO 2222, GBBC 2040, MK16, D s0432-1, RNS 08.23.3.1A) exhibited high cellulase activity while compared to three other strains (IFB0158, IFB0221, IFB0223). All the tested strains, except for IFB0223, were able to produce proteases (Figure 3B). The strains RNS 08.23.3.1A, IFB0099, IFB0221, MK16, and D s0432-1 showed the highest protease activity.

The swimming ability of the strains was also analyzed in low concentration agar medium (Figure 3C). The mobility of the *D. solani* strains was variable and the relationship between motility and virulence was not evident. However, the reduced swimming capacity of strain IFB0223 matched with its low virulence. In contrast, strains IFB0099 with the best swimming capacity in the agar medium showed the greatest ability to macerate potato tuber tissue (Figures 2, 3C).

In our further analyzes of the relationship between virulence, phenotypic traits and genomic signatures, we focused on the comparison of two strains IFB0099 and IFB0223 exhibiting extremely different levels of virulence, with IFB0099 still showing the highest virulence and IFB0223 the lowest virulence, regardless of the experimental conditions.

### The *D. solani* genome structure

For 14 analyzed genomes, the number of scaffolds, the number of N bases added to the genome sequence during the assembly process, the genome size, and the GC content are reported in Table 1. The number of scaffolds ranged from 1 (5 genomes, namely IFB0099, IFB0223, IPO 2222, GBBC 2040, RNS 08.23.3.1A) to 38 (IFB0221). The length of these *D. solani* genomes varied from 4,860,047 to 4,985,571 bp for the strains GBBC 2040 and RNS 05.1.2A, respectively. Their GC content ranged from 56.34 to 56.13% for the strains GBBC 2040 and RNS 05.1.2A, respectively (Table 1).

**Figure 4:**
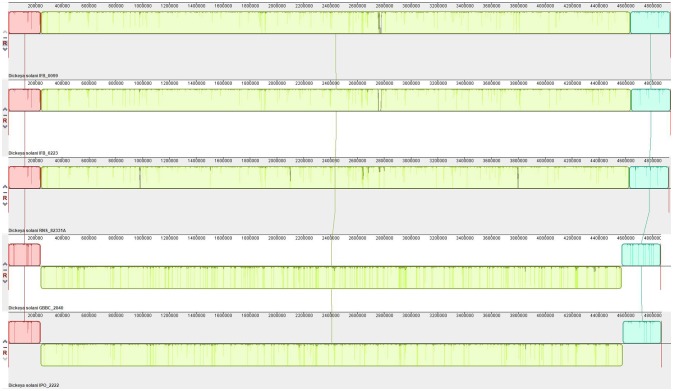
Synteny of the *D. solani* genomes. Pairwise alignments of genomes were generated by Mauve 2.4.0 (development snapshot Mauve_2015_02_26). Inside each block a similarity profile of the genomic sequence is presented. Height of the similarity profile corresponds to the average level of genomic conservation in that region. Areas that are completely white were not aligned and probably contain sequence elements specific to a particular genome. Height of the similarity profile is inversely proportional to the average alignment column entropy over a region of alignment.

Synteny analysis was performed on 5 genomes successfully closed to one scaffold and showed no strong rearrangements among these *D. solani* strains (Figure 4). There are only 3 syntenic blocks, with two inversions in the case of the strains IFB0099, IFB0223, and RNS 08.23.3.1A, in comparison to GBBC 2040 and IPO 2222. With the Prokka annotation, the number of total predicted genes varied from 4,273 for the strain GBBC 2040 to 4,536 for RNS 08.23.3.1A (Table 2). The number of protein encoding genes varie between 4,138 for the strain RNS 07.7.3B and 4,303 for RNS 05.1.2A (Table 2). The overall ANI analysis of the 14 *D. solani* strains showed low genomic variability between them, with ANI values ranging from 98.60 to 99.99% (Supplementary Table 1).

**Table 2:** Genome and pangenome contents of *D. solani* strains.

	**Total number of genes**	**Genome**				**Pangenome^a^**		
***D. solani*** **genome**		**Predicted number of genes encoding**				**Core genes**	**Accessory genes**	**Unique genes**
		**Proteins**	**rRNAs**	**tRNAs**	**tmRNA**			
IFB0099	4,326	4164	22	75	1	3,756	387	21
IFB0158	4,289	4149	4	64	1	3,756	384	9
IFB0221	4,283	4148	4	60	1	3,756	383	9
IFB0223	4,328	4167	22	75	1	3,756	382	29
NCPPB4479^T^ IPO 2222	4,327	4199	3	62	1	3,756	429	14
GBBC 2040	4,273	4145	3	62	1	3,756	368	21
MK10	4,376	4245	3	64	1	3,756	356	133
MK16	4,288	4157	3	64	1	3,756	380	21
D s0432-1	4,331	4167	22	74	1	3,756	385	26
RNS 08.23.3.1A	4,536	4151	18	72	1	3,756	380	15
PPO 9019	4,280	4146	1	64	1	3,756	380	10
PPO 9134	4,282	4144	3	65	1	3,756	384	4
RNS 05.1.2A	4,436	4303	1	64	1	3,756	175	372
RNS 07.7.3B	4,274	4138	1	64	1	3,756	376	6

a *Solely genes encoding proteins were included in the analysis*.

### The *D. solani* pangenome

**Figure 5:**
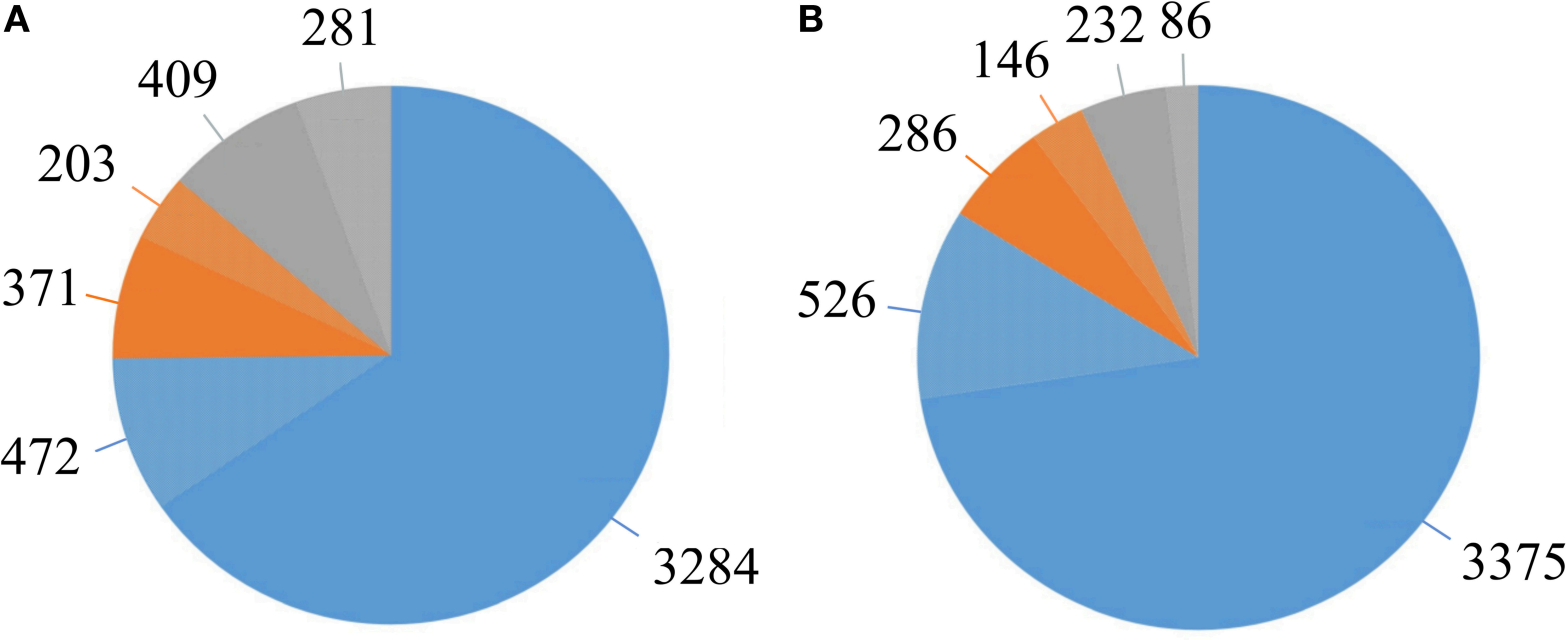
The *D. solani* pangenome shape. **(A)** Pangenome shape based on the number of genes in all 14 strains. **(B)** Pangenome shape after exclusion of strain RNS 05.1.2A. Blue—represents the core genome. Orange—represents the accessory genome. Gray—represents the unique genome. Lighter shades of each color represent the fractions of genes annotated as hypothetical proteins.

The pangenome of the selected 14 strains contains 5,020 protein encoding genes with a core genome of 3,756 genes (74.8%) and an accessory genome of 574 genes (11.5%) (Table 2, Supplementary Table 2). The number of unique genes, as calculated by Mauve, was 690 (13.7%) (Figure 5A, Table 2) and 281 of them were annotated as encoding hypothetical proteins. The accessory genome fraction contains from 356 to 429 genes of the strains MK10 and IPO 2222, respectively, with a notable exception of the strain RNS 05.1.2A which has only 175 genes in this fraction (Table 2). The unique genome fraction varies from only 4 genes for the strain PPO 9134 to 372 genes for RNS 05.1.2A. Twelve strains possess from 4 to 29 unique genes. Interestingly, MK10 and RNS 05.1.2A were the most diverse strains with 133 and 372 unique genes, respectively (Table 2). If the most diverse strain RNS 05.1.2A is excluded from the pangenome analysis, the pangenome is reduced to 4,651 genes, the core genome increases up to 3,901 genes, the number of accessory genes decreases to 432 and the number of unique genes goes down to only 318 (Figure 5B).

### Conservation of virulence and regulatory genes and repertoire of prophages in the *D. solani* genomes

Because of their involvement in the soft-rot symptoms, we verified the presence of genes encoding PCWDEs and their regulators in 14 *D. solani* genomes. All the genes encoding PCWDEs previously described in *D. dadantii* (pectate lyases, pectin methylesterases, cellulases, or proteases) are found in the *D. solani* core genome. Each gene has an identical sequence in all the 14 *D. solani* strains. In the case of pectate lyases, protein sequence identities between *D. solani* and *D. dadantii* 3937 reache 98% for PelB, 97% for PelC and PelZ, 96% for PelA, PelE, PelW and PelX, 95% for PelL and PelN, 94% for PelD and 84% for PelI. For the proteases and cellulase, the identities level between *D. solani* and *D. dadantii* 3937 reache 98% for PrtA and PrtB, 95% for PrtG and 93% for CelZ.

The genes encoding transcriptional regulators of virulence genes, previously identified in *D. dadantii* 3937 (such as KdgR, PecS, PecT, CRP, Fur, Fis, H-NS), are also well conserved and found in the *D. solani* core genome. These regulators are very similar to the corresponding proteins of *D. dadantii* 3937, with protein identity of 100% for CRP, Fis and H-NS, 99% for KdgR and Fur, 98% for PecS, and 92% for PecT. As for PCWDEs, their protein sequences are identical among the *D. solani* strains.

**Figure 6:**
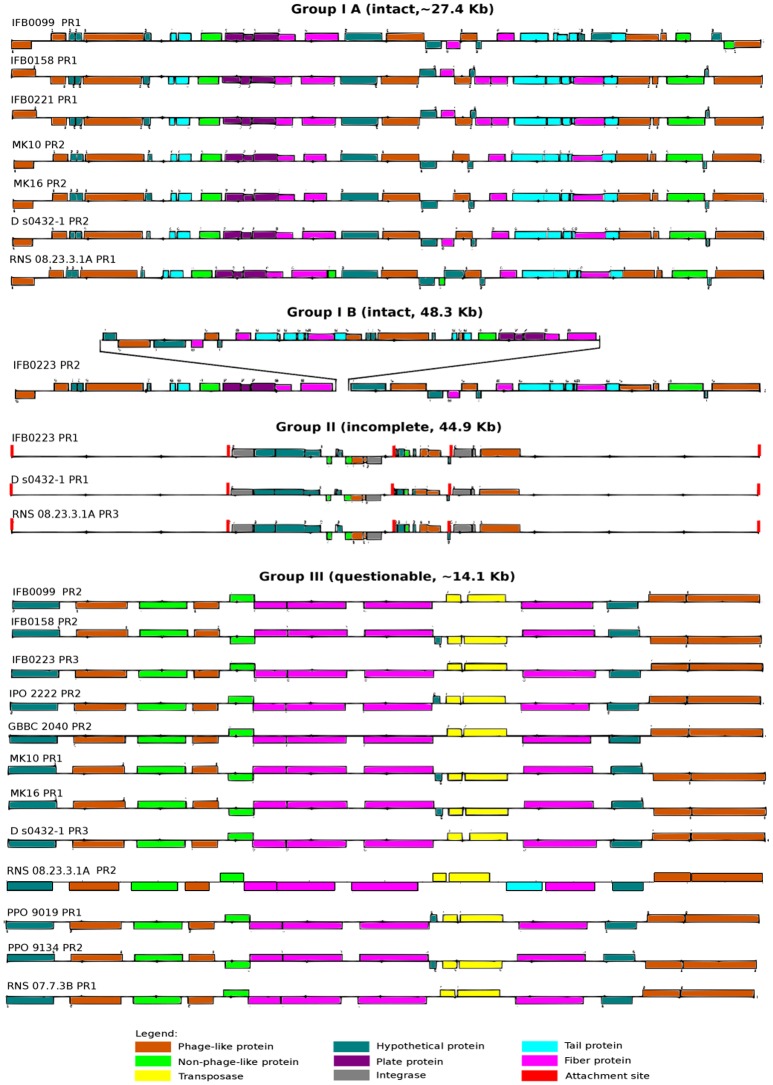
Prophages present in the *D. solani* genomes. Prophages are divided into three groups: intact (IA and IB), incomplete (II) and questionable (III) on the basis of structural analysis based on data provided by PHAST on-line web server (http://phast.wishartlab.com/; Zhou et al., 2011).

Due to their importance in genome evolution, the presence of prophages was analyzed in the 14 *D. solani* genomes. Of 35 predicted prophages, 23 could be gathered into three main groups, I, II and III, according to their sequence similarity (Figure 6, Table 3). The sequence of the 12 other prophages greatly varied and they were assigned as ungrouped or “n” (Supplementary Figure 1, Table 3). In addition, each prophage was predicted as intact, questionable or incomplete. All group I prophages were predicted as intact, group II prophages were described as incomplete, and group III prophages as questionable (Figure 6). Interestingly, each genome contains at least one member of each defined group I, II, and III.

**Table 3:** Prophages present in the analyzed *D. solani* genomes.

***D. solani* strain**	**No. of the prophage sequence**	**Length (kb)**	**Sequence completeness**	**Positions in the *D. solani* genome**	**Group^a^**
IFB0099	PR1 PR2	27 14.1	intact questionable	2748142-2775153 4672375-4686494	I A III
IFB0158	PR1 PR2	27.4 14.1	intact questionable	637761-665201 3565525-3579644	I A III
IFB0221	PR1 PR2	27.4 14.1	intact questionable	637761-665201 4859798-4874569	I A N
IFB0223	PR2 PR1 PR3	48.3 44.9 14.1	intact incomplete questionable	2744993-2793313 432810-477786 4677009-4691128	I B II III
NCPPB4479^T^ IPO 2222	PR1 PR2	16 14.1	incomplete questionable	2913332-2929416 4611857-4625976	n III
GBBC 2040	PR1 PR2	26.9 14.1	incomplete questionable	2905276-2932269 4604558-4618677	n III
MK10	PR2 PR1	27.2 14.1	intact questionable	2756228-2783473 159129-173256	I A III
MK16	PR2 PR1	27.2 14.1	intact questionable	2687323-2714567 159136-173255	I A III
D s0432-1	PR2 PR1 PR3	27.4 44.9 14.1	intact incomplete questionable	2732844-2760284 435970-480946 4643974-4658093	I A II III
RNS 08.23.3.1A	PR1 PR3 PR2	28.3 44.9 14.5	intact incomplete questionable	649811-678182 3258604-3303580 2561220-2575779	I A II III
PPO 9019	PR1	14.1	questionable	1622153-1636272	III
PPO 9134	PR1 PR2	12.3 14.1	questionable questionable	965401-977759 4823112-4837231	n III
RNS 05.1.2A	PR4 PR5 PR6 PR1 PR2 PR7 PR3	23.5 34 31.5 11.4 15.2 19.4 20.8	intact intact intact incomplete incomplete incomplete questionable	2062173-2085685 2397356-2431394 4377687-4409244 522314-533731 871014-886304 4482742-4502177 1782074-1802942	n n n n n n n
RNS 07.7.3B	PR1 PR2	14.1 13.1	questionable questionable	1644970-1659089 3091451-3104564	III n

a *Unique prophage sequences were marked with ‘n'*.

The prophage composition of each strain varies quantitatively and qualitatively among the 14 *D. solani* strains (Table 3). The prophage number varies from a single prophage for the strain PPO 9019 (group III) to seven prophages for RNS 05.1.2A, all attributed to “n” because of their diverse structures (three intact, three incomplete and one questionable prophage). For majority of *D. solani* genomes, i.e. nine, two prophages are predicted. Only four bacterial strains (IFB0099, IFB0158, MK10 and MK16) share the same prophage repertoire (group I and III). Three strains (IFB0223, RNS 08.23.3.1A and D s0432-1) have three predicted prophages in their genomes and a similar prophage repertoire (group I, II, and III).

Based on our classification, group I contains eight intact prophages, found in seven virulent strains (sub-classified in group IA). The gene structure is very similar, with a prophage length varying from 27 kb (strain IFB0099) to 28.3 kb (strain RNS 08.23.3.1A). The eighth group I prophage (sub-classified in group IB) is found in the weakly virulent strain IFB0223. Despite its size of 48.3 kb, it has a similar basal structure. Indeed, prophages of groups IA and IB differ by a deletion/insertion of about 20 kb in the middle of their sequence (Figure 6). The three prophages of group II, found in three genomes, are 44.9 kb long. Group III comprises 12 questionable prophages of 14.1 kb, found in 12 genomes. Prophages not classified in these three groups (“n”) are predicted to be intact (3 prophages), or questionable (4 prophages) or incomplete (5 prophages) (Supplementary Figure 1). It should be noted that many phage-related genes are classified in the unique genome fraction, demonstrating their genetic diversity.

### The *D. solani* panregulon

**Figure 7:**
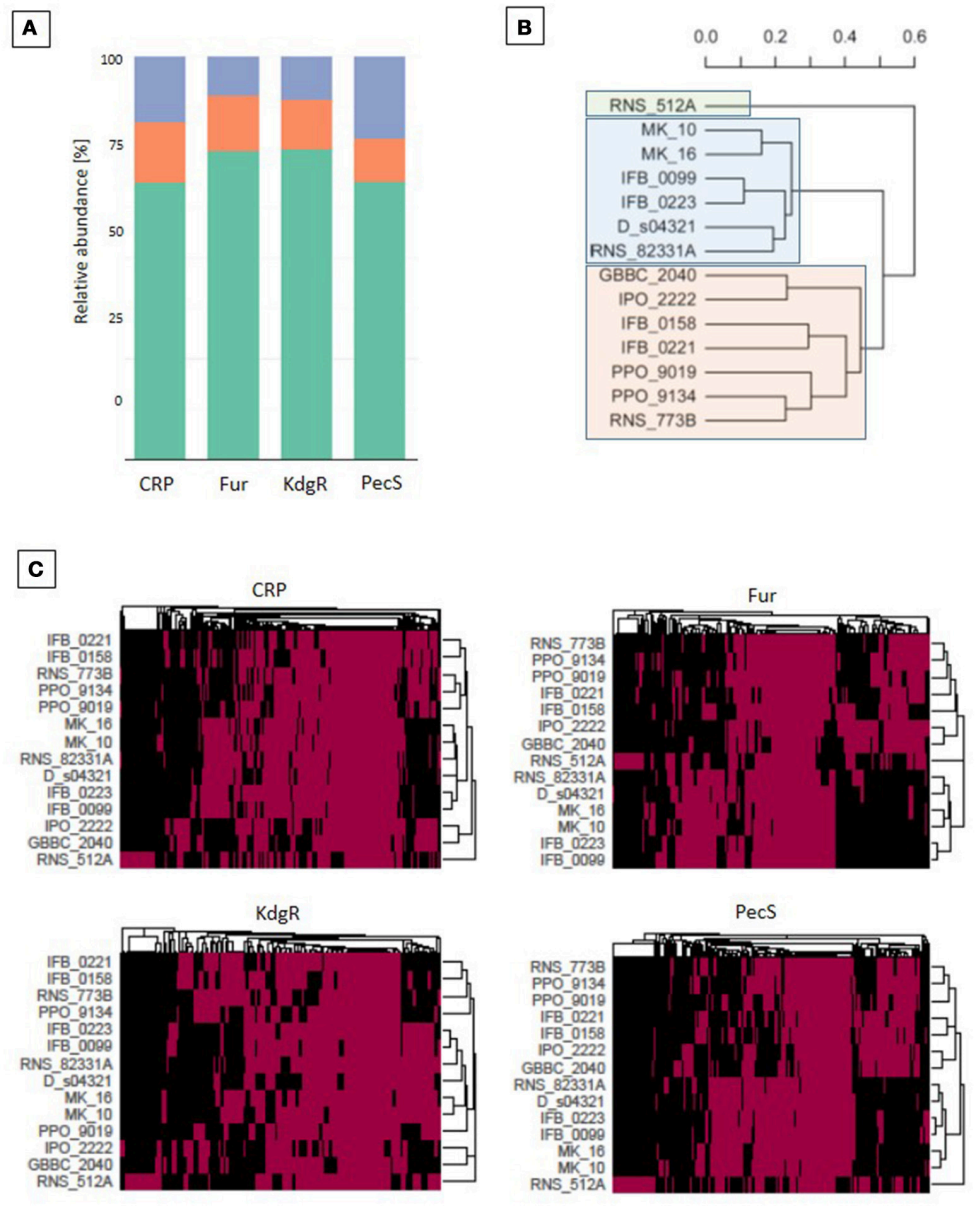
Regulon analysis based on binding sites predictions. Predictions of the transcription regulators (CRP, Fur, KdgR, and PecS) binding sites were made with the use of MAST version 4.10.1. (Bailey and Elkan, 1994) and the version 1.62b of the *Bio.motifs* package from the Biopython library (Cock et al., 2009). **(A)** The regulon representation for the 4 transcription regulators (TFs): in orange for core, green for accessory, violet for unique TF targets. **(B)** Phylogenetic tree based on the total regulons predicted for the 4 TFs. The UPGMA methods on Jaccard distance were utilized for phylogenetic analysis. **(C)** Heatmaps for CRP, Fur, KdgR and PecS TFs, which represent all predicted genes that are putatively regulated by the selected TF. A red box indicates the presence whereas a black one indicates the absence of a TF binding site in the upstream region of the gene.

In order to provide additional genomic information that can help to interpret the differences in virulence observed between *D. solani* strains, a prediction of the regulons of TFs known to be involved in *Dickeya* pathogenesis was performed. Large putative regulons were found for the major regulators: CRP, Fur, KdgR, and PecS. All strains contain a set of shared target genes, constituting the core regulon (binding sites present in all the genomes), but also a dispensable regulon fraction (Figure 7A). CRP has the largest predicted panregulon with 807 potential targets, of which 121 are in the core regulon, 554 in the accessory regulon and 132 in the unique regulon (Figure 7A). The predicted PecS panregulon includes 784 targets of which 84 are in the core regulon, 540 in the accessory regulon and 160 in the unique regulon. The Fur predicted panregulon has 174 targets, of which 24 are present in the core regulon, 133 in the accessory regulon and 17 in the unique regulon. KdgR has the smallest predicted panregulon with 130 targets, of which 16 are in the core regulon, 100 in the accessory regulon and 14 in the unique regulon (Figure 7A).

The *D. solani* strains grouped into three main clusters by considering the differential occurrence of all four TF targets (Figure 7B). The strain RNS 05.1.2A groups separately from other 13 *D. solani* strains, which form two clusters, one of 6 strains (MK10, MK16, IFB0099, IFB0223, D s0432-1 and RNS 08.23.3.1A) and the other of 7 strains (GBBC 2040, IPO 2222, IFB0158, IFB0221, PPO 9019, PPO 9134, and RNS 07.7.3B). The heatmaps show phylogenetic relationships of the strains based on TF binding sites present/absent in the regulatory region of the target genes (Figure 7C). Clustering was similar when considering each single TF or the phylogenetic model based on all four TFs. Small rearrangements of the tree contents are visible only in case of CRP and KdgR.

**Figure 8:**
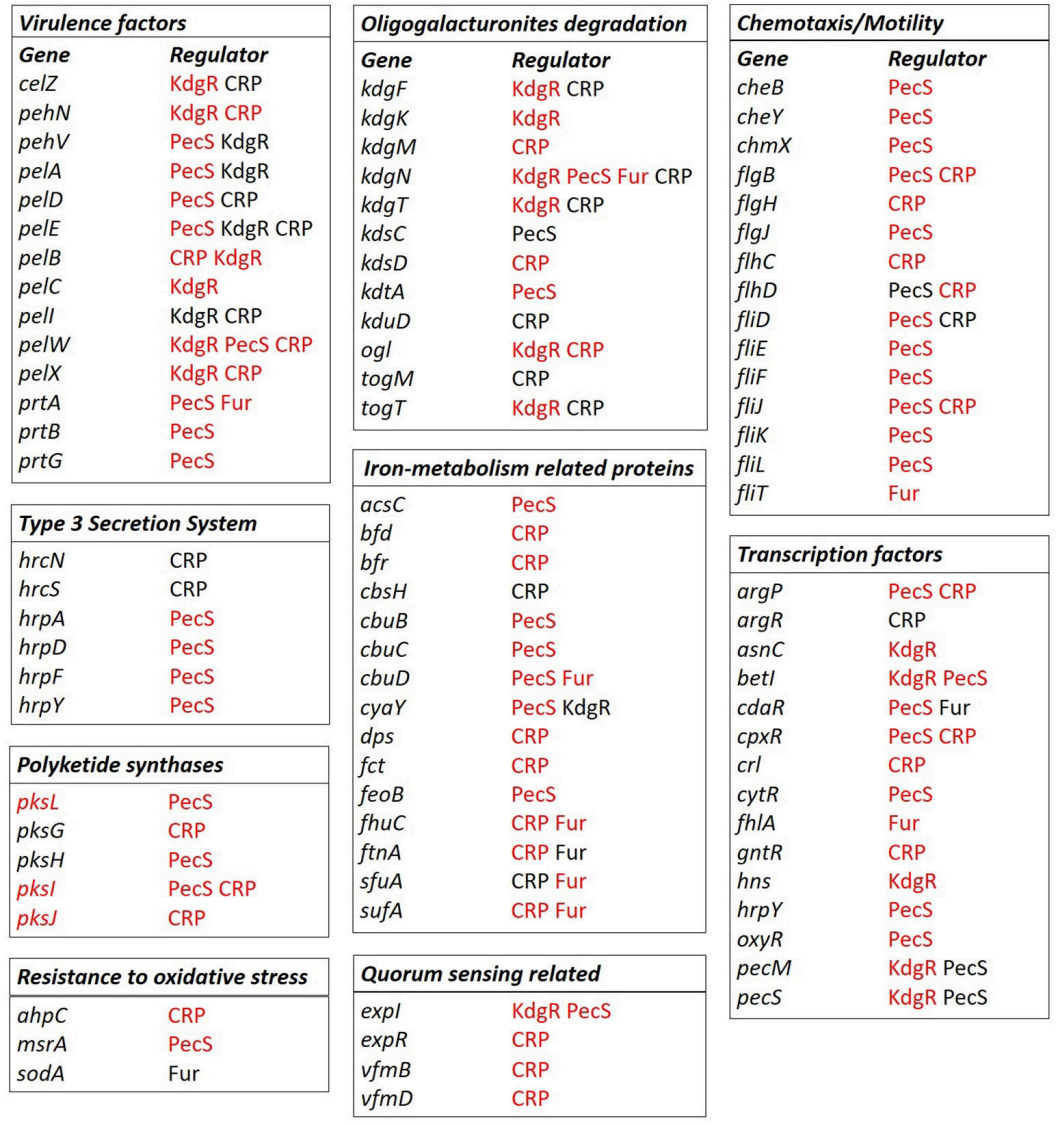
Selected regulons of *D. solani* based on binding sites predictions. Predictions of the CRP, Fur, KdgR, and PecS (TFs) binding sites were conducted with the use of MAST version 4.10.1. (Bailey and Elkan, 1994) and *Bio.motifs* package from Biopython library (version 1.62b) (Cock et al., 2009) for the virulence factors: proteins involved in pectin and oligogalacturonide degradation, chemotaxis, and motility, iron metabolism, polyketide synthesis, resistance to oxidative stress, transcription factors, quorum sensing-related proteins and T3SS components. Gene names are marked in black when the genes belong to the core genome and in red when the genes belong to the accessory genome fractions. The TFs name is given in black to indicate the core regulon, and in red to indicate the accessory regulon.

In more detail, the predicted CRP core and accessory regulon contains several genes involved in cellulose and pectin degradation such as *celZ, pehN, pelB, pelC, pelD, pelE, pelI, pelW, pelX, kdgF, kdgM, kdgN, kdgT, kdsD, kduD, ogl, togM*, and *togT* (Figure 8). The core or accessory regulon predicted for CRP also includes genes related to flagella (*flgB, flgJ, flhC, flhD, fliD, fliJ)*, T3SS (*hrpN* and *hrpS*), polyketide synthesis (*pksG, pksI*, and *pksJ*), iron metabolism (*bfd, bfr, cbsH, dps, fct, fhuC, ftnA, sfuA* and *sufA*) and resistance to oxidative stress (*ahpC*). Interestingly, the predicted CRP regulon contains some genes constituting the two quorum sensing systems described for *D. dadantii* 3937, namely *expR, vfmB* and *vfmD*. CRP was also predicted to regulate other TFs, such as *argP, argR, cpxR, crl*, and *gntR* (Figure 8).

As expected, the predicted Fur regulon contains mostly genes related to iron assimilation, namely *cbuD, fhuC, ftnA*, and *sufA* (Figure 8). It also includes a protease gene (*prtA*), a gene involved in oligogalacturonide transport (*kdgN*), a flagellar gene (*fliT*), a gene involved in resistance to oxidative stress (*sodA*) and TF genes, such as *cdaR* and *fhlA* (Figure 8).

The predicted KdgR core or accessory regulon includes several genes involved in cell wall component degradation and catabolism: *kdgF, kdgK, kdgN, kdgT, kduI, ogl, togT, celZ, pehN, pehV, pelA, pelB, pelC, pelE, pelI, pelW, pelX, togT*, (Figure 8) and other genes previously shown to be regulated by KdgR in *D. dadantii* (*ppsA, pykF, sotA, spiX, tpgX, ydiA, ygjV, chmX*) (Rodionov et al., 2004). More surprisingly, it contains several genes involved in regulation, which were not previously identified in the *D. dadantii* KdgR regulon (*cyaY, expI, hns, asnC, betI, pecS*) (Figure 8).

The predicted PecS core and accessory regulon contains genes encoding PCWDEs (*pehV, pelA, pelD, pelE, pelW, prtA, prtB*, and *prtG*), genes related to chemotaxis and motility (*cheB, cheY, chmX, flgB, flgJ, flhD, fliD, fliE, fliF, fliJ, fliK*, and *fliL*), T3SS (*hrpA, hrpD, hrpF* and *hrpY*) (Figure 8), polyketide synthesis (*pksL, pksH*, and *pksI*), iron metabolism (*acsC, cbuB, cbuC, cbuD, cyaY*, and *feoB*) and quorum sensing (*expI*). The predicted PecS regulon also includes other genes encoding TFs, such as *argP, betI, cdaR, cpxR, cytR*, and *oxyR*.

In summary, genes coding for PCWDEs and important for oligogalacturonide degradation are predicted to be regulated mainly by KdgR, PecS and CRP. Chemotaxis and motility related genes as well as genes encoding T3SS proteins and polyketide synthases are predicted to be regulated by CRP and PecS. Genes significant for iron-metabolism are predicted to be regulated by Fur, but also by PecS and CRP. Quorum sensing related genes seem to be regulated by CRP, KdgR and PecS. However, it should be underlined that for all regulon predictions the acquired hits are putative, and consequently, they provide only starting points for later investigations.

### Genomic background of the differences in virulence between *D. solani* IFB0099 and IFB0223

This study performed on 14 *D. solani* strains showed that they have homogenous genomes despite different phenotypic features, not only in their ability to macerate plant tissue but also in pectinolytic, cellulolytic, and proteolytic activities and motility. In order to elucidate the genomic background of these differences, we concentrated our analysis on two *D. solani* strains: IFB0099 and IFB0223, which differ significantly in virulence level on potato (Figure 2A) and chicory (Potrykus et al., 2014).

All genes encoding PCWDEs and their regulators are present in both genomes and their sequences indicated 100% identity. *D. solani* IFB0099 exhibited a significantly higher activity of pectinolytic and cellulolytic enzymes than IFB0223 (Figures 2B, 3A). The strain IFB0223, which showed no protease production, possesses genes coding for three proteases: PrtA, PrtB, and PrtG and their T1SS secretion system, and all of these genes show 100% identity with those of *D. solani* IFB0099.

At the genome level, IFB0099 and IFB0223 are highly similar to each other. There are only 38 genes in IFB0099 genome that are not present in IFB0223. Inversely, 41 genes are found in the IFB0223 genome but absent in the IFB0099 genome. In both cases, most of these genes are prophage related. *D. solani* IFB0099 genome contains one intact prophage of group IA (27 kb) and one questionable prophage (about 14 kb) (Figure 6). IFB0223 contains one intact prophage of group IB (48.3 kb), one incomplete prophage (about 45 kb) and one questionable prophage (about 14 kb) (Figure 6). The long intact prophage of IFB0223 contains two copies of several genes: *smrA* (endonuclease), *pcaK (*4-hydroxybenzoate transporter), *hin* (invertase), *cas1 (*clavaminate synthase 1) and 16 other phage related genes. Moreover, the long intact prophage of IFB0223 (group IB, 48.3 kb) is homologous to the group IA prophage of strain FB0099, localized in the same genomic region but 27 kb long.

Apart from differences in the prophage related genes, IFB0223 lacks the genes encoding a peptidoglycan transglycosylase (*mtgA*), an isoprenoid biosynthesis protein with amidotransferase-like domain (*elbB*), a T3SS component (*hrpQ*) and a miscRNA called ArcZ, while these genes were present in all other *D. solani* genomes.

When looking into the nucleotide sequence similarity of the protein coding genes present in the genomes of both IFB0099 and IFB0223, only 10 SNPs were found and 5 of them determine non-synonymous variations. For instance, a SNP in *rpoB* caused a change from Ser in IFB0099 to Phe in IFB0223, and the SNP in *dmlR_13* replaced Ser in IFB0099 with a Pro in IFB0223. More surprisingly, we observed 3 non-synonymous substitutions (2 × Ala → Thr and Ser → Ala) in the *fhaB*1 gene (IFB0099_02260) between the two strains. The *fha* genes encode large filamentous hemagglutinins secreted outside the cells. These FHA proteins may facilitate the attachment of the bacterial cells to their host. According to Prokka annotation, two *fhaB* genes are found in the genome of IFB0223 (IFB0223_02255 and IFB0223_02334) and three *fhaB* genes are present in the genome of IFB0099 (IFB0099_02260, IFB0099_02339 and IFB0099_02340). Both genomes contain *fhaB*1 (IFB0099_02260, IFB0223_2255), localized closely to the gene *dadA* coding for the *d*-amino acid dehydrogenase small subunit. Despite the 3 non-synonymous substitutions observed in their genes, the FhaB1 proteins of IFB0099 and IFB0223 possess a signal peptide of the same length (4165 amino acids) and a similar domain structure, as predicted by hmmscan against Pfam database (Finn et al., 2015). Thus, both FhaB1 proteins are supposed to be functional, even if they might show different efficiencies.

A second gene, *fhaB*2 (IFB0223_02334 and IFB0099_02339) is predicted in both genomes, closely to the region coding for the harpin HrpN. The *fhaB*2 gene present in the IFB0223 genome (IFB0223_02334, 13,791 bp) is homologous to the gene *hecA* described in the *D. chrysanthemi* strain EC16 (Rojas et al., 2002; Finn et al., 2015). Both proteins are similar in length and structure (predicted by hmmscan) and both possess a signal peptide, which may suggest that the IFB0223 FhaB2 protein is functional. In contrast, two successive shorter genes (IFB0099_02339, 6,525 bp and IFB0099_02340, 7,242 bp) are predicted in the corresponding position in the genome of IFB0099, and only one of them encodes a protein with a signal peptide. Comparison of the nucleotide sequences of the genomic regions coding for *fhaB*2 in IFB0223 and IFB0099 showed high similarity between them with only 2 differences in the 13,791 nucleotide long alignment. One of these differences leads to a synonymous substitution at position 6,504 of the alignment (C → T). More interestingly, at position 7,235 of the alignment, the deletion of a G nucleotide leads to a frameshift, giving rise to an Opal stop codon (TGA) at position 7,240 in the IFB0099 sequence. Because of this deletion, the translated protein is shorter in IFB0099 (IFB0099_02340) than in IFB0223, with 2,413 amino acids instead of 4,596. A third *fhaB* is then predicted in IFB0099 (IFB0099_02339); however this truncated protein has no signal peptide.

## Discussion

In the present study, we combined the results obtained from the pangenome and the predicted panregulon to explore the background of diverse virulence levels of *D. solani* strains. First, we developed a genome assembly pipeline for *D. solani* which allowed us to close the genomes of a highly virulent strain, IFB0099, and a low virulent strain, IFB0223 (Figure 1). Reads originating from only one sequencing technology (PacBio) are sufficient for closing the genome. Instead of a set of contigs, we obtained a fully annotated chromosome without N-bases. Importantly, the software used is freely available and no home-made scripts were utilized during the assembly and annotation, making the pipeline easy to follow by other researchers.

### High homogeneity of *D. solani* genomes and its effect on the pangenome structure

Genomes of 14 *D. solani* strains were explored in order to disclose some explanation for their diverse ability to macerate plant tissue (Tsror et al., 2013; Potrykus et al., 2014, 2016, 2018; Golanowska et al., 2017). Our comparative study, performed on 10 available and 4 newly sequenced *D. solani* genomes, confirmed the high genomic homogeneity in this species, even among strains isolated from soft-rotting plants or rhizosphere of healthy plants. The homogeneity of *D. solani* genomes is reflected by ANI values of about 99.9, with the exception of strain RNS 05.1.2A which gives values around 98.7. As noticed by Khayi et al. (2015), the strain RNS 05.1.2A forms a specific sub-group among *D. solani* strains. The *D. solani* genomes have a similar structure, revealed by their high synteny presented for five successfully closed genomes. The genes encoding major virulence determinants (pectinases, cellulases, and proteases) and their regulators (KdgR, PecS, PecT, Fis, H-NS, CRP, and Fur) shared 100% identity.

For the newly sequenced genomes, the number of predicted protein encoding genes varied from 4,148 (IFB0221) to 4,167 (IFB0223), with 60 to 75 genes encoding tRNAs. These results are comparable to those obtained for other *D. solani* strains (Garlant et al., 2013; Khayi et al., 2014, 2015, 2016; Pédron et al., 2014). Considering the 14 *D. solani* genomes, their mean size is 4,892,047 bp and their mean GC content is 56.23%. These 14 genomes were used for description of the species pangenome, with the following repartition of the genes: 74.8% into the core genome and 25.2% into the dispensable (accessory and unique) genome. If strain RNS 05.1.2A is excluded, the core genome increases by 4%, the accessory genome decreases by 25% and the number of unique genes decreases by about 50%. The pangenome of *D. solani* appears open and, after adding another genome to the pool of analyzed genomes, the unique genome fraction increases on average by 50 genes (by 25 genes if strain RNS 05.1.2A is excluded).

Pangenome analysis is a good way to describe bacterial lifestyle and to explain differences in the strain pathogenicity. The genes found in the dispensable genome fraction can be responsible for the strains ability to survive in rare or specific conditions. The dispensable genome of *D. solani* comprised only 25.2% of the genes and it mainly consisted of prophage-related genes. In contrast, recent data using *Pectobacterium parmentieri* strains showed that the dispensable genome of this more heterologous species comprises about 49% of the genes (Zoledowska et al. unpublished results).

### Variability in prophage sequences present in *D. solani* genomes

A notable variability in the prophage sequences present in *D. solani* genomes is for the first time reported in this study. The *D. solani* strains generally contain two to three predicted prophages, among them: intact, incomplete and questionable ones. Most of these prophages belong to 3 groups but different repertoires are observed depending on the strain (Table 3). The presence of the prophage sets found in individual strains may be related to their origin, for example two strains isolated in Poland (IFB0099 and IFB0158) have the same prophage repertoire (Figure 6 and Table 3). The presence of similar prophages in the strains isolated in Scotland and Israel (MK10 and MK16) can be explained by the fact that majority of potato seeds imported by Israel are produced in Scotland (Figure 6 and Table 3). Strain RNS 05.1.2A contains the highest number of predicted prophages and all of them are found only in this strain. This confirms the report of Khayi et al. (2015) indicating that the strain RNS 05.1.2A forms a separate clade in the *D. solani* phylogenetic tree (Khayi et al., 2015). It could be speculated that strain RNS 05.1.2A does not possess strong tools to protect itself from phage infections.

The role of prophages in bacteria can be variable. Temperate bacteriophages are particularly important agents of horizontal gene transfer. They could be vectors of new virulence factors or/and toxins that may change a non-virulent strain into a pathogenic one (Varani et al., 2013). Prophage insertion could also have structural genomic impacts (Varani et al., 2013). Since chromosome organization is known to influence transcriptional networks in *D. dadantii* (Jiang et al., 2015), the expression of some genes, in *D. dadantii* and in *D. solani*, could be affected by modification of genom structure due to prophage insertion.

### Analysis of *D. solani* panregulon, effect of regulators on virulence genes

Panregulon analysis is a powerful tool, complementary to comparative genomics, that helps to identify new targets for known TFs and to elucidate roles they play in microorganisms. We used TF binding site prediction tools to identify putative targets of four TFs, namely CRP, KdgR, PecS, and Fur in *D. solani* genomes. Each of them plays a crucial role in *Dickeya* virulence. However, until now, these regulators have been analyzed almost exclusively in the *D. dadantii* model strain 3937. Thus, it is not known whether the data obtained in strain 3937 are relevant in other *Dickeya* species and, even, in other *D. dadantii* strains. Originality of our approach relies on analysis the corresponding regulons in a set of *D. solani* strains. The binding site predictions allowed us to identify the *D. solani* panregulon for KdgR, PecS, CRP and Fur, and to differentiate the core and accessory regulons. For each TF, the core regulon contains from 10.7 to 15% of the predicted regulated genes. Thus, majority of the predicted targets, 85–89.7%, are in the accessory regulon. Many genes encoding virulence factors, such as PCWDEs and proteins involved in pectin degradation are found in the predicted accessory regulons of KdgR, PecS, and CRP. For instance, the pectate lyase gene *pelE* seems to be regulated by KdgR and CRP in all the tested *D. solani* strains, but only some of them are regulated by PecS (Figure 7). Thus, some virulence genes could have differential expression among *D. solani* strains.

KdgR is primarily a repressor of genes encoding pectinases and pectin catabolism. In *D. dadantii* 3937, more than 50 genes are induced in the presence of pectin and KdgR directly controls at least 13 operons (Hugouvieux-Cotte-Pattat and Robert-Baudouy, 1989; Hugouvieux-Cotte-Pattat et al., 1996). Comparative genomics of different *Enterobacteriaceae* predicted that KdgR could control as many as 32 operons, including several novel targets such as *chmX, dhfX, gntB, ppsA pykF, spiX, sotA, tpfX, yeeO*, and *yjgK* (Rodionov et al., 2004). In our study, we also found in the *D. solani* KdgR panregulon all the KdgR targets reported in *D. dadantii* 3937. In addition, we found novel putative members of the KdgR regulon, such as *pir, gacA, uvrY, ybbH, nagC, expI, hns, nadR, asnC, betI, pecS, pecM*. Function of these KdgR targets are not limited to pectin degradation and some of them are involved in regulation. For instance, GacA regulates T3SS at a post transcriptional level in *D. dadantii* 3937 (Yang et al., 2008). The *expI* gene encodes a homoserine lactone synthase involved in quorum sensing. Interactions between these two regulators could have an impact on the virulence of *D. solani*.

In *D. dadantii*, PecS acts as a repressor of genes encoding PCWDEs. The strongest repression is exerted on the genes encoding secreted proteins (*pelC, pelE, pelL, pelN, celZ, prtABC, nipE, virK, avrL*) and on the genes involved in the biosynthesis of excreted compounds, such as the blue pigment indigoidine (*indABC*), a surfactant (*rhlA*) and the quorum sensing signal VFM (*vfmZ, vfmE, vfmAB*) (Hommais et al., 2008). In addition to these genes, we also found protease genes in the PecS accessory regulon of *D. solani*. We previously noticed that a *pec*S mutant of IFB0223 has a de-repressed protease activity in comparison to the wild-type strain (Potrykus et al., 2014). In *D. dadantii* 3937, PecS also regulates genes involved in the flagellum biogenesis (*fliE* and *fliFGHIJKLMNOPQR*) (Rouanet et al., 2004). In our study, putative binding sites for PecS were predicted in the regulatory regions of several *D. solani* flagellum genes (*fliE, fliD, fliF, fliJ, fliK, fliL*). It was previously observed that *pecS* inactivation in *D. solani* resulted in variable phenotypes regarding swimming motility; the *pecS* mutants of 2 strains, among the 4 tested, were hypermotile (Potrykus et al., 2014). Transcriptomic analysis of *D. dadantii pecS* mutants showed that PecS directly or indirectly controls from 400 to more than 600 genes (Hommais et al., 2008; Pédron et al., 2018), placing PecS at the top of a major regulatory cascade. In *D. solani*, the predicted PecS panregulon contains approximately 780 genes, including other TFs like CRP, KdgR and Fur. Considering either KdgR or PecS, our regulon prediction seems credible and can be used with confidence to investigate bacterial regulatory networks.

The main role of CRP is to control assimilation of carbohydrates but it is also involved in various other cellular processes. As expected, the CRP regulon of *D. solani* includes several pectate lyase genes and genes involved in pectin catabolism. It also contains genes involved in mobility, T3SS, toxin production, iron metabolism regulation (Figure 8). Among them, we observed two genes *vfmB* and *vfmD* which are involved in the biosynthesis of a *Dickeya* specific communication signal of yet unknown structure (Nasser et al., 2013; Potrykus et al., 2018). This specific quorum sensing system influences the PCWDE production in *D. solani*, as well as in *D. dadantii* (Nasser et al., 2013; Potrykus et al., 2018).

PecT is an interesting case of a TF sensitive to DNA conformation changes (Hérault et al., 2014) and it has an important role in regulation of the PCWDE genes in *D. solani* (Potrykus et al., 2014). Unfortunately, prediction of PecT regulon on the basis of *in silico* analysis was not possible since the PecT binding site has no defined DNA sequence.

### Genomic background related to differences in virulence between *D. solani* IFB0099 and IFB0223

In order to find information on virulence differences in the genomic background, we concentrated our analysis on strains IFB0099 and IFB0223 which differ in their virulence level on potato (Figure 2A) and chicory (Potrykus et al., 2014) and in addition their activity of PCWDE (Figures 2B, 3A,B). However, sequences encoding proteins involved in cell wall degrading indicated 100% homology.

When comparing these two strains, most strain-specific genes are prophage related. The genomes of IFB0099 and IFB0223 contain only one intact prophage of 27 kb and 48.3 kb, respectively, localized in the same genome region (Figure 6). The IFB0223 intact prophage contains two copies of 20 genes (*smrA, pcaK, hin, cas1* and prophage related genes), suggesting a duplication event. The presence of a longer prophage could indirectly contribute to the expression of virulence genes in IFB0223 through changing the spatial organization of this DNA region. Some TFs involved in *Dickeya* virulence modulate their activity in function of the DNA conformation, as shown for PecT (Hérault et al., 2014) or even KdgR (Bouyioukos et al., 2016).

Concerning the phage elements encoded in the *D. solani* genomes, only weakly virulent IFB0223 possesses an additional phage genetic material (intact phage from group IB) which is twice as large as the phage occurring in the corresponding regions of IFB0099 and other virulent *D. solani* strains. The potential role of prophages in the variability of *D. solani* virulence will need further investigation.

In comparison to IFB0099 and all other virulent strains, IFB0223 lacks the genes *mtgA, elbB, hrpQ* and a gene encoding a miscRNA called ArcZ. ArcZ belongs to a family of small regulatory RNAs (sRNAs) which are important post-transcriptional regulatory components in bacteria. In *Erwinia amylovora* ArcZ participates in the positive control of T3SS, amylovoran exopolysaccharide production, biofilm formation, and motility (Zeng and Sundin, 2014). ArcZ is also responsible for the translational activation of RpoS in response to oxidative stress in *E. coli* (Mandin and Gottesman, 2010). These indications suggest that ArcZ could play a role in *D. solani* pathogenicity and its absence might be responsible for low virulence of IFB0223.

Following Prokka annotation 30 genes coding for filamentous hemagglutinins (adhesin, CdiA, FhaB) were found in the 14 *D. solani* genomes. Each *D. solani* strain possesses at least one (IPO 2222, GBBC 2040, MK10), two (IFB0158, IFB0221, IFB0223, PPO 9134, RNS 05.1.2A, RNS 07.7.3B) or even three predicted adhesin genes (IFB0099, MK16, D s0432-1, RNS 08.23.3.1A, PPO 9019). The *fhaB* genes annotated in *D. solani* genomes vary in length and code for proteins of as little as 246 (MK16) to as many as 4,596 (IFB0223) amino acids. The FhaB proteins have similar domain structure. The most variable region found in CdiA/FhaB is the cytotoxic domain after a common pre-toxin VENN motif. Since these proteins are highly variable, the phylogenetic relationship of the *D. solani* 21 cytotoxin domains extracted from adhesin proteins was examined (Supplementary Figure 2, Supplementary Table 3). Two FhaB proteins described for *E. coli* EC93 (CdiA) and *E. chrysanthemi* EC16 (HecA) were included in the analysis. Interestingly, majority of the cytotoxic domains extracted from *D. solani* adhesins form two distinct clades A and B (Supplementary Figure 2). Sequences from clade B are similar to HecA from *E. chrysanthemi* EC16, which exhibit a Dnase activity. Moreover, both types of cytotoxic domains are encoded by most of the *D. solani* genomes (except for IPO 2222, GBBC 2040, MK10, which have only one *fhaB* gene, Supplementary Table 2).

Genomic data suggested that strain IFB0223 produces two functional secreted adhesins, FhaB1 and FhaB2. These two adhesins could be responsible for its efficient adherence ability, but also might limit its mobility. In contrast, disruption of the gene *fhaB2* in IFB0099 may cause a loss of function for the adhesin FhaB2 and less efficient attachment of IFB0099 to plant surfaces. Poor attachment may result in an increased cell mobility that, finally, could contribute to higher virulence of this strain. Such hypothesis resembles observations of Ionescu et al. (2014) who showed that a highly virulent mutant of *X. fastidiosa* has lower attachment to the xylem vessels than the wild type strain. Therefore, strong attachment of bacteria to plant surfaces could restrict their movement and their capacity to colonize the plants and to systemically spread within the plant host. Strain IFB0223 appeared significantly less mobile than strain IFB0099 (Figure 3). Khayi et al. (2015) underlined that non-synonymous variations in the flagellar genes *fliC* and *fliN* correlate with the absence of motility and weak virulence of the *D. solani* strain 3296. In the case of low and highly virulent strains, IFB0223 and IFB0099, flagellar genes and flagella motor genes (*motA* and *motB*) have identical DNA sequences. Thus, low mobility of IFB0223 could rather be related to a high attachment capacity.

## Conclusions

The genome-wide comparison based on pangenome and panregulon analysis of 14 *D. solani* genomes, showed only few differences between the highly virulent potato isolate *D. solani* IFB0099 and the low virulent rhizosphere isolate *D. solani* IFB0223. In contrast, these two strains differ significantly in the production of virulence factors like pectinases, cellulases and proteases, and in their mobility. Their genomes diverge in the number and size of prophages. Only IFB0223 lacks the genes encoding peptidoglycan transglycosylase (*mtgA*), a T3SS component (*hrpQ*) and a miscRNA called ArcZ, a small regulatory RNA which can provide resistance to oxidative stress. Another relevant difference is the disruption of the gene *fhaB2*, encoding adhesin, in strain IFB0099 and other virulent *D. solani* strains. Inactivation of this gene may result in less efficient attachment of bacteria to plant surfaces and, concurrently, to increased mobility bacteria within plant intercellular spaces. The more adhesive but less motile strain IFB0223 showed weaker aggressiveness than the spreading faster and causing more severe disease symptoms strain IFB0099.

## Author contributions

MalG conducted the majority of the experiments, analyzed, and interpreted the pangenome and panregulon data and prepared the first draft of the manuscript. MP and AM-P contributed to complete genomes, prepared figures and tables, discussed results, and contributed to the writing of the final version of the manuscript. MK made the pangenome bioinformatics analysis. GB made the panregulon bioinformatics analysis. MarG performed preliminary pangenome analysis and contributed to the writing of the manuscript. MB, IM, and KS critically revised the final version of the manuscript. EL, NH-C-P, and AM conceived the idea, designed research and critically revised the final version of the manuscript. All the authors read and approved the manuscript.

### Conflict of interest statement

The authors declare that the research was conducted in the absence of any commercial or financial relationships that could be construed as a potential conflict of interest.
