# Supplementary material for: Comparison of Highly and Weakly Virulent Dickeya solani Strains, With a View on the Pangenome and Panregulon of This Species
Source: Front Microbiol. 2018 Aug 31;9:1940. doi: 10.3389/fmicb.2018.01940 (PMC6127512; doi:10.3389/fmicb.2018.01940)
Supplement: Supplementary file 1 [file Table_1.docx]

**Supplementary Table 1. ANI values for *D. solani* and *D. dadantii* 3937 genomic sequences.**

| ***D. solani* strains** | **IFB0099** | **IFB0158** | **IFB0221** | **IFB0223** | **NCPPB4479^T^ IPO 2222** | **GBBC 2040** | **MK10** | **MK16** | **D s0432-1** | **RNS 08.23.3.1A** | **PPO 9019** | **PPO 9134** | **RNS 05.1.2A** | **RNS 07.7.3B** |
| --- | --- | --- | --- | --- | --- | --- | --- | --- | --- | --- | --- | --- | --- | --- |
| **IFB0099** | - | 99.99 | 99.99 | 99.98 | 99.92 | 99.77 | 99.97 | 99.98 | 99.99 | 99.99 | 99.89 | 99.93 | 98.77 | 99.95 |
| **IFB0158** | 99.99 | - | 99.98 | 99.98 | 99.91 | 99.76 | 99.95 | 99.99 | 99.98 | 99.98 | 99.90 | 99.90 | 98.74 | 99.94 |
| **IFB0221** | 99.99 | 99.98 | - | 99.98 | 99.91 | 99.76 | 99.95 | 99.97 | 99.97 | 99.98 | 99.91 | 99.90 | 98.75 | 99.95 |
| **IFB0223** | 99.98 | 99.98 | 99.98 | - | 99.91 | 99.75 | 99.97 | 99.97 | 99.98 | 99.98 | 99.90 | 99.92 | 98.75 | 99.94 |
| **NCPPB4479^T^ IPO 2222** | 99.92 | 99.91 | 99.91 | 99.91 | - | 99.81 | 99.91 | 99.92 | 99.92 | 99.92 | 99.84 | 99.85 | 98.74 | 99.89 |
| **GBBC 2040** | 99.77 | 99.76 | 99.76 | 99.75 | 99.81 | - | 99.76 | 99.76 | 99.76 | 99.75 | 99.71 | 99.70 | 98.60 | 99.74 |
| **MK10** | 99.97 | 99.95 | 99.95 | 99.97 | 99.91 | 99.76 | - | 99.96 | 99.97 | 99.98 | 99.88 | 99.90 | 98.74 | 99.93 |
| **MK16** | 99.98 | 99.99 | 99.97 | 99.97 | 99.92 | 99.76 | 99.96 | - | 99.98 | 99.99 | 99.92 | 99.93 | 98.75 | 99.95 |
| **D s0432-1** | 99.99 | 99.98 | 99.97 | 99.98 | 99.92 | 99.76 | 99.97 | 99.98 | - | 99.94 | 99.92 | 99.91 | 98.73 | 99.94 |
| **RNS 08.23.3.1A** | 99.99 | 99.98 | 99.98 | 99.98 | 99.92 | 99.75 | 99.98 | 99.99 | 99.94 | - | 99.92 | 99.93 | 98.75 | 99.95 |
| **PPO 9019** | 99.89 | 99.90 | 99.91 | 99.90 | 99.84 | 99.71 | 99.88 | 99.92 | 99.92 | 99.92 | - | 99.83 | 98.72 | 99.87 |
| **PPO 9134** | 99.93 | 99.90 | 99.90 | 99.92 | 99.85 | 99.70 | 99.90 | 99.93 | 99.91 | 99.93 | 99.83 | - | 98.75 | 99.87 |
| **RNS 05.1.2A** | 98.77 | 98.74 | 98.75 | 98.75 | 98.74 | 98.60 | 98.74 | 98.75 | 98.73 | 98.75 | 98.72 | 98.75 | - | 98.83 |
| **RNS 07.7.3B** | 99.95 | 99.94 | 99.95 | 99.94 | 99.89 | 99.74 | 99.93 | 99.95 | 99.94 | 99.95 | 99.87 | 99.87 | 98.83 | - |
| ***D*. *dadantii* 3937** | 94.07 | 94.08 | 94.01 | 94.11 | 94.02 | 93.99 | 94.09 | 94.06 | 94.10 | 94.02 | 94.06 | 94.05 | 94.14 | 94.15 |

ANI (%) are shown. *D. dadantii* 3937 was incorporated as an interspecies reference. OrthoANIu algorithm (Yoon *et al*., 2017) was utilized.
