# Supplementary material for: Comparison of Highly and Weakly Virulent Dickeya solani Strains, With a View on the Pangenome and Panregulon of This Species
Source: Front Microbiol. 2018 Aug 31;9:1940. doi: 10.3389/fmicb.2018.01940 (PMC6127512; doi:10.3389/fmicb.2018.01940)
Supplement: Supplementary file 3 [file Table_3.DOCX]

**Supplementary Table 3.** Hmmerscan analysis of chosen protein sequences against Pfam, Tigram, Superfamily databases of the *Dickeya solani* proteins annotated as filamentous hemagglutinin genes within *D. solani* genomes (Prokka). The protein sequences of genes chosen for analysis were annotated as filamentous hemagglutinin FhaB. As a reference three protein sequences of adhesins from *Escherichia coli,* *Dickeya chrysanthemi* and *Xylella fastidiosa* were included in the analysis.

| **Strain** | **IFB collection** | **ORF number of predicted FhaB proteins** | **Protein length** | **Hemagglutinin repeats (Pfam)** | **Adhesion hecA repetitive motifs (Tigram)** | **TPS secretion domain (Superfamily)** | **Pre-toxin domain with VENN motif** | **Cytotoxic domain^a^** |
| --- | --- | --- | --- | --- | --- | --- | --- | --- |
| ***Dickeya solani* strains** | | | | | | | | |
| **IFB0099** | Yes | 2339 | 2174 | 2 | 0 | 0 | 1 | A |
|  |  | 2340 | 2413 | 15 | 41 | 0 | 0 | none |
|  |  | 2260 | 4165 | 16 | 29 | 1 | 1 | B |
| **IFB0158** | Yes | 637 | 2282 | 9 | 0 | 0 | 1 | B |
|  |  | 3919 | 1900 | 6 | 0 | 0 | 1 | A |
| **IFB0221** | Yes | 637 | 2316 | 9 | 0 | 0 | 1 | B |
|  |  | 3837 | 2108 | 5 | 0 | 0 | 1 | A |
| **IFB0223** | Yes | 2334 | 4596 | 19 | 41 | 1 | 1 | A |
|  |  | 2255 | 4165 | 17 | 30 | 1 | 1 | B |
| **IPO2222** | Yes | 1912 | 1111 | 2 | 0 | 0 | 1 | A |
| **GBBC 2040** | Yes | 1893 | 1111 | 2 | 0 | 0 | 1 | A |
| **MK10** | Yes | 2388 | 1071 | 3 | 0 | 0 | 1 | A |
| **MK16** | Yes | 2238 | 483 | 3 | 0 | 1 | 0 | none |
|  |  | 2317 | 1142 | 2 | 0 | 0 | 1 | A |
|  |  | 4286 | 246 | 2 | 7 | 0 | 0 | none |
| **D s0432-1** | Yes | 2271 | 2133 | 9 | 0 | 0 | 1 | B |
|  |  | 2272 | 802 | 3 | 0 | 0 | 0 | none |
|  |  | 2273 | 2174 | 4 | 4 | 0 | 1 | A |
| **RNS 08.23.3.1A** | Yes | 382 | 4165 | 17 | 30 | 1 | 1 | B |
|  |  | 461 | 4128 | 18 | 40 | 0 | 1 | A |
|  |  | 462 | 1368 | 4 | 18 | 1 | 0 | none |
| **PPO 9019** | No | 1783 | 2174 | 5 | 0 | 0 | 1 | A |
|  |  | 2691 | 1576 | 11 | 34 | 0 | 0 | none |
|  |  | 2696 | 929 | 4 | 19 | 0 | 0 | none |
| **PPO 9134** | No | 1033 | 2174 | 5 | 0 | 0 | 1 | A |
|  |  | 1421 | 1588 | 10 | 34 | 0 | 0 | none |
| **RNS 05.1.2A** | No | 386 | 1902 | 5 | 0 | 0 | 1 | A |
|  |  | 2802 | 2185 | 7 | 0 | 0 | 1 | C |
| **RNS 07.7.3B** | No | 2589 | 1576 | 11 | 34 | 0 | 0 | none |
|  |  | 3465 | 2174 | 5 | 0 | 0 | 1 | A |
| ***D. chrysanthemi* EC16** | Yes | AAN38709.1  (HecA) | 3848 | 14 | 30 | 1 | 1 | Dnase |
| ***E. coli* EC93** | No | Q3YL96  (CdiA) | 3132 | 11 | 15 | 1 | 1 | Pore forming |
| ***Xylella fastidiosa*** | No | KGM20939.1 | 2504 | 0 | 0 | 0 | 0 | 0 |

^a^ Activity shown when it was already proved (Aoki et al. 2010). Letters A, B, C, designate different cytotoxic domains that can be differentiated between *D. solani* proteins on the basis of phylogenetic analysis presented in **Supplementary Figure 2**.
