# Supplementary figures and images for: Comparison of Highly and Weakly Virulent Dickeya solani Strains, With a View on the Pangenome and Panregulon of This Species
Source: Front Microbiol. 2018 Aug 31;9:1940. doi: 10.3389/fmicb.2018.01940 (PMC6127512; doi:10.3389/fmicb.2018.01940)

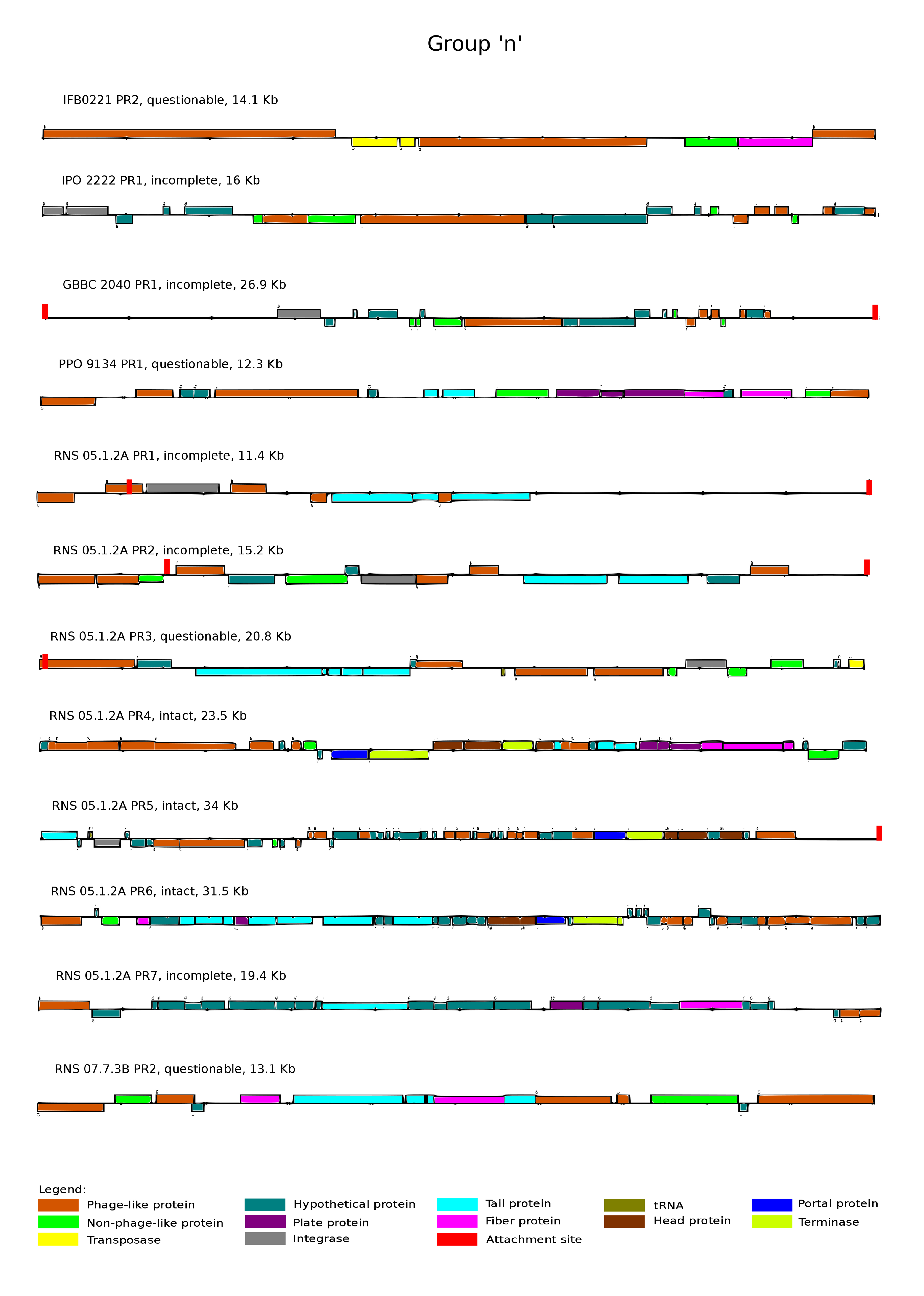

Supplement: Supplementary file 4 [file Image_1.JPEG]

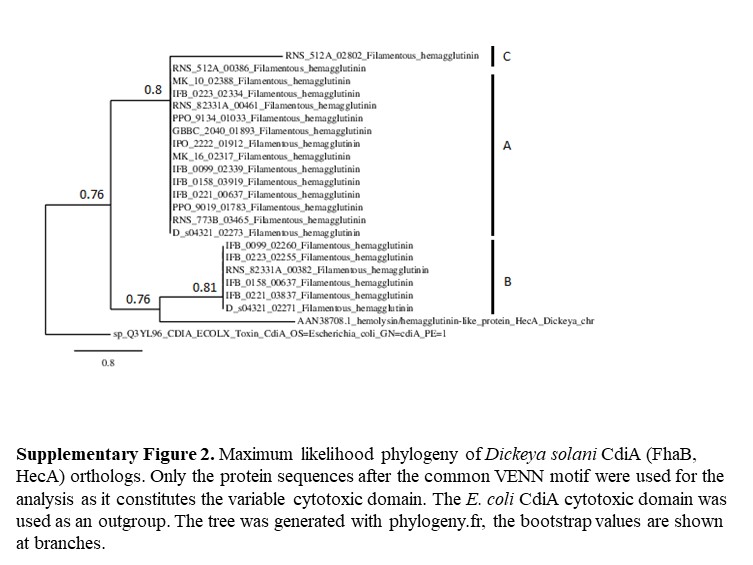

Supplement: Supplementary file 5 [file Image_2.JPEG]
